# Evaluation of the Skin Sensitization Potential of Carbon Nanotubes Using Alternative In Vitro and In Vivo Assays

**DOI:** 10.3390/toxics8040122

**Published:** 2020-12-16

**Authors:** Sung-Hyun Kim, Dong Han Lee, Jin Hee Lee, Jun-Young Yang, Hyo-Sook Shin, JeongPyo Lee, Kikyung Jung, Jayoung Jeong, Jae-Ho Oh, Jong Kwon Lee

**Affiliations:** Division of Toxicological Research, National Institute of Food and Drug Safety Evaluation, Ministry of Food and Drug Safety, Osong, Cheongju 28159, Chungcheongbuk-do, Korea; donghan04@korea.kr (D.H.L.); tod98@korea.kr (J.H.L.); yangjy@korea.kr (J.-Y.Y.); aqua978@korea.kr (H.-S.S.); origene@korea.kr (J.L.); kikyung@korea.kr (K.J.); 0jjy@korea.kr (J.J.); chopin68@korea.kr (J.-H.O.)

**Keywords:** skin sensitization, alternative to animal testing, KeratinoSens™, LLNA, nanomaterial, CNT

## Abstract

Carbon nanotubes (CNTs) are one of the major types of nanomaterials that have various industrial and biomedical applications. However, there is a risk of accidental exposure to CNTs in individuals involved in their large-scale production and in individuals who use products containing CNTs. This study aimed to evaluate the skin sensitization induced by CNTs using two alternative tests. We selected single-wall carbon nanotubes and multi-walled carbon nanotubes for this study. First, the physiochemical properties of the CNTs were measured, including the morphology, size, and zeta potential, under various conditions. Thereafter, we assessed the sensitization potential of the CNTs using the ARE-Nrf2 Luciferase KeratinoSens™ assay, an in vitro alternative test method. In addition, the CNTs were evaluated for their skin sensitization potential using the LLNA: BrdU-FCM in vivo alternative test method. In this study, we report for the first time the sensitization results of CNTs using the KeratinoSens™ and LLNA: BrdU-FCM test methods in this study. This study found that both CNTs do not induce skin sensitization. These results suggest that the KeratinoSens™ and LLNA: BrdU-FCM assay may be useful as alternative assays for evaluating the potential of some nanomaterials that can induce skin sensitization.

## 1. Introduction

Carbon nanotubes (CNTs) are a major type of nanomaterial that is used for various industrial and biomedical applications [1,2]. In recent years, with the growing number and production volume of CNTs, concerns about their toxicity have also increased exponentially. Generally, nanomaterials are defined as particles less than 100 nm in at least one dimension [3], which exhibit various physicochemical properties associated with a nanostructure [4].

The various physicochemical characteristics of a nanomaterial are the major determinants of its toxic potential [5,6]. In normal environmental conditions, nanomaterials are mostly poorly soluble; however, some nanomaterials have shown to be soluble in lysosomal fluid or gastric fluid [7,8]. Dissolution of nanomaterials can cause toxicity due to the release of ions [9].

The major exposure pathways of nanomaterials are inhalation, ingestion, and absorption into the skin. Absorption pathways within the skin can cause lesions, such as local inflammation, contact allergy, and skin sensitization [10,11]. Recently, with an exponential increase in the cosmetic commercialization of nanomaterials and the safety concerns associated with them, the safety evaluation of nanomaterials has gained importance [12]. In addition, in recent cosmetic tests, the importance of alternative test methods is increasing due to concern about animal welfare and the 3R principles [13,14]. However, as these guidelines are based on chemical substances, it is necessary to develop alternative test methods that reflect the properties of nanomaterials.

The current knowledge on the chemical and biological mechanisms associated with skin sensitization has been summarized in the form of an adverse outcome pathway, starting with the molecular initiating event through intermediate events to the adverse effect, namely allergic contact dermatitis [15]. The skin sensitization test, adverse outcome pathway (AOP), is largely classified into an animal test and nonanimal test method. Nonanimal test methods include the direct peptide reactivity assay (DPRA, key event 1) to confirm peptide reactivity, the ARE-Nrf2 Luciferase Test (KeratinoSens™, key event 2), and the human Cell Line Activation Test (h-CLAT, key event 3) assay to evaluate the sensitization of test substances using cell lines. Key event 4 (LLNA: DA, BrdU-ELISA, and FCM) is an animal test method to evaluate the activation of mice lymph nodes to sensitizers [16,17,18,19].

According to a recent report, sensitization evaluation using several nanomaterials was performed, and the applicability of these assays for testing the nanomaterials was evaluated [20,21]. However, there is still a lack of information on the skin sensitization results of nanomaterials. Therefore, this study was performed to evaluate the skin sensitization potential of two types of CNTs using the ARE-Nrf2 Luciferase KeratinoSens™ and LLNA: BrdU-FCM assays.

## 2. Materials and Methods

### 2.1. Carbon Nanotubes

Single-wall carbon nanotubes (SWCNTs, product No. 704121) and multi-walled carbon nanotubes (MWCNTs, product No. 698849) were purchased from Sigma-Aldrich (St Louis, MO, USA). Their morphology was observed by transmission electron microscopy (TEM) (JEM-1200EX II, JEOL, Tokyo, Japan) and the average diameter was calculated by measuring both CNTs using the ImageJ software program ver.1.48. The zeta potential of the CNTs was measured using a Zetasizer-Nano ZS instrument (Malvern Instruments, Malvern, UK) in different working solutions: Dulbecco’s modified Eagle’s medium (DMEM; GIBCO, Grand Island, NY, USA) containing 1% heat-inactivated fetal bovine serum (FBS; GIBCO) and *N*,*N*-dimethylformamide (DMF; Sigma-Aldrich, CASRN. 68-12-2) solution containing 3% heat-inactivated mouse serum. The levels of endotoxin were evaluated using an Endpoint Chromogenic Limulus Amoebocyte Lysate assay (Cambrex, Walkersville, MD, USA).

### 2.2. Preparation of CNT Suspensions

The suspensions of CNTs in media were prepared by slightly modifying a previously described method [22,23]. Briefly, the CNT stock solutions were dispersed in distilled water (DW) and sonicated at 40 kHz with a 100 W output power for 30 min in a bath-type sonicator (Saehan-Sonic, Seoul, Korea). Thereafter, DMEM supplemented with 1% FBS was added to different working concentrations. As CNTs cannot be converted into molar concentration, as determined in the test guideline 442D, the test concentration was set based on the mass dose (µg/mL). In addition, the test concentration was set through two preliminary tests to confirm the EC50 concentration of CNTs. In LLNA: BrdU-FCM assay, CNT stock solution was dispersed in DW and sonicated at 40 kHz with a 100 W output power for 30 min in a bath-type sonicator (Saehan-Sonic). Thereafter, a 3% serum equivalent of the final volume was added to the initial dispersion and further dispersed for 30 min. Finally, DMF solution was added to prepare a working solution (25, 50, and 100%).

### 2.3. Cell Culture

A transgenic keratinocyte cell line, with a stable insertion of the Luciferase reporter gene under control of the ARE-element KeratinoSens™ cells, was provided from Givaudan Suisse SA (Vernier, Switzerland). KeratinoSens™ cells were cultured in DMEM media supplemented with 10% FBS and 0.5 mg/mL Geneticin (G418; Sigma-Aldrich, St. Louis, MO, USA). The cells were sub-cultured every 3–4 days at 80–90% confluence for a maximum of 25 passages. For the assay, KeratinoSens™ cells were seeded into a 96-well culture plate at a density of 1 × 10^4^ cells/well. Then, the culture media were replaced with fresh medium (DMEM supplemented with 1% FBS) and incubated in a humidified atmosphere condition of 5% CO_2_ at 37 °C.

### 2.4. CNT Treatments and KeratinoSens™ Assay Methods

KeratinoSens™ cells were seeded into 96-well plates at a density of 1 × 10^4^ cells/well and incubated overnight to reach approximately 80% confluency. The cells were washed once with pre-warmed DPBS (Gibco), followed by the addition of fresh medium containing single-wall carbon nanotubes (SWCNTs) and multi-walled carbon nanotubes (MWCNTs) (0.05–1000 µg/mL), and the plates were then incubated for 48 h. Positive control (cinnamic aldehyde, 4–64 µM) was tested in parallel. The viability of the treated cells was measured using the thiazolyl blue tetrazolium bromide (3-(4,5-dimethylthiazo-2-yl)-2,5-diphenyl-tetrazolium bromide) assay reduction test (Promega, Madison, WI, USA). To exclude colorimetric interference from CNTs present in the cells, the supernatant was transferred into clear 96-well plates and the absorbance was measured at 570 nm with a microplate reader (Tecan, Männedorf, Switzerland). The cell viability (%) was calculated based on the optical density of the vehicle control and blank. Luciferase activity was measured using the One-Glo™ Luciferase assay kit (Promega). The luminescence intensity of each sample was measured using a luminometer (Promega) and multi-microplate reader (Synergy 2, BioTek, Winooski, VT, USA). Luciferase induction was calculated based on the luminescence values of the vehicle control and blank.

### 2.5. Animals

Female BALB/C mice (7 weeks old, Specific Pathogen Free) were purchased from ORIENT BIO Inc. (Seongnam, Korea). Animals were kept at an animal facility in the Korea Ministry of Food and Drug Safety (MFDS) and acclimated for at least six days before experiments. Mice were housed in a relative humidity of 3-C70% at 22 ± 3 °C. This experiment was approved by the Institutional Animal Care and Use Committee (IACUC) of MFDS (Approval number: MFDS-20-013c2; date: 23 April 2020).

### 2.6. CNTs Treatments and LLNA: BrdU-FCM Assay Methods

On days 1, 2, and 3, dispersed CNT suspension, vehicle, and positive control (25% hexyl cinnamic aldehyde in AOO) were applied to the dorsal skin of each ear of the mouse at the same time-point. The CNT suspensions were prepared fresh daily before application. On day 5, the mice were intraperitoneally injected with 100 μL of BrdU solution (20 mg/mL). On day 6, the mice were sacrificed, and their auricular lymph nodes were excised. Then, excised lymph nodes were mashed with a spatula to prepare lymph node cells (LNCs). Isolated LNCs were counted using a hemocytometer after staining with trypan-blue solution. The counted LNCs (1.5 × 10^6^ cells/mL) were prepared, according to the protocol provided in the manufacturer’s kit. The viable LNCs were counted and a total of 10,000 gated cells were analyzed using BD FACS Calibur^TM^ flow cytometry (BD Biosciences, San Jose, CA, USA), as previously described [24,25]. Stimulation index (SI) values were calculated using the formula, as described in the OECD TG 442B guideline. If the SI value was 2.7 or above, the test materials were classified as sensitizers.

## 3. Results

### 3.1. Physicochemical Characteristic of CNTs

**Figure 1:**
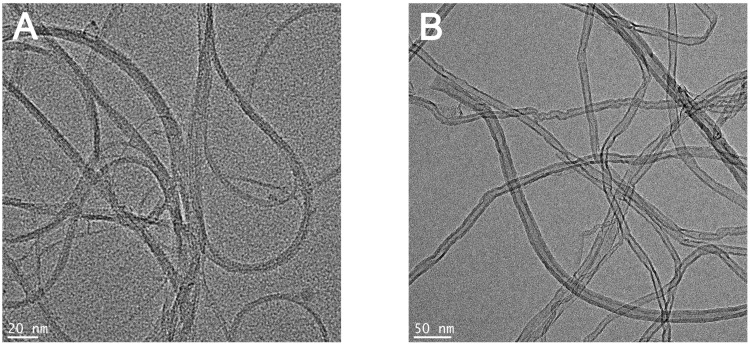
Transmission electron microscopy images of the (**A**) single-wall carbon nanotubes (SWCNTs) (bar = 20 nm) and (**B**) multi-walled carbon nanotubes (MWCNTs) (bar = 50 nm) in distilled water.

Figure 1 shows the TEM images of SWCNTs and MWCNTs used in this study. The results confirmed that both SWCNTs and MWCNTs had a size of less than 100 nm in one dimension. The characteristics of CNTs are summarized in Table 1. Measurement of the zeta potential showed that all CNTs were negatively charged, with charge in distilled water (DW) and working solution. The results of the Limulus Amoebocyte Lysate test showed that both SWCNTs and MWCNTs had endotoxin levels that were lower than the limit of detection (0.1 U/mL).

### 3.2. Evaluation of CNTs in the KeratinoSens™ Assay

**Figure 2:**
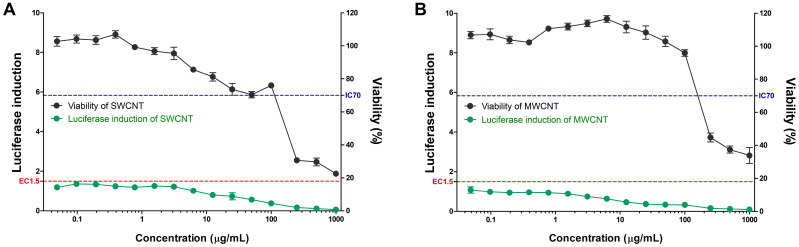
The induction of luciferase activity (green line) and cell viability (black line) in the KeratinoSens^TM^ assay. KeratinoSens^TM^ cells were treated with the (**A**) SWCNTs and (**B**) MWCNTs. Data are expressed as mean ± standard deviation values (*n* = 6).

**Figure 3:**
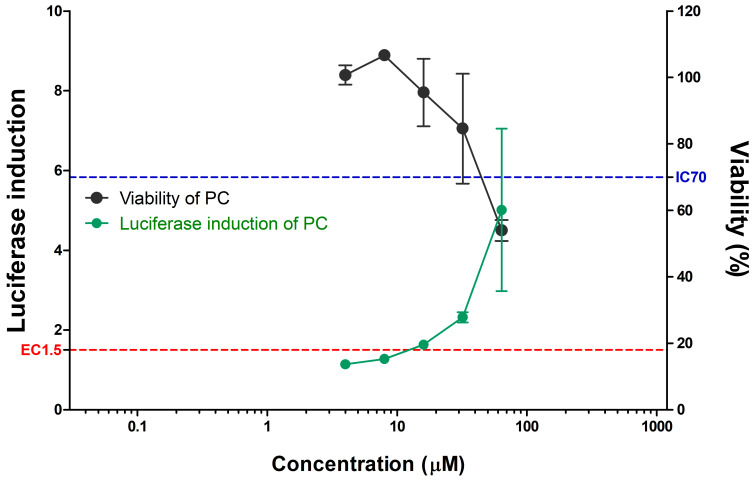
Luciferase activity (green line) and cell viability (black line) of positive control (cinnamic aldehyde, CASRN. 14371-10-9) in KeratinoSens™ assay. Data are expressed as mean ± standard deviation values (*n* = 6). Positive control (4–64 µM) was tested in parallel.

SWCNTs and MWCNTs were assessed for their skin sensitization potential using the KeratinoSens^TM^ assay (Table 2 and Figure 2). All CNTs did not induce the activity of the luciferase reporter in contrast to the positive control (Figure 2 and Figure 3). The EC_1.5_ value for both CNTs was >1000 µg/mL, thus classifying it as a nonsensitizer. Cytotoxicity, IC_50_ values were found to be 234.98 µg/mL for MWCNTs and 185.90 µg/mL for SWCNTs.

### 3.3. Evaluation of CNTs in the LLNA: BrdU-FCM Assay

**Figure 4:**
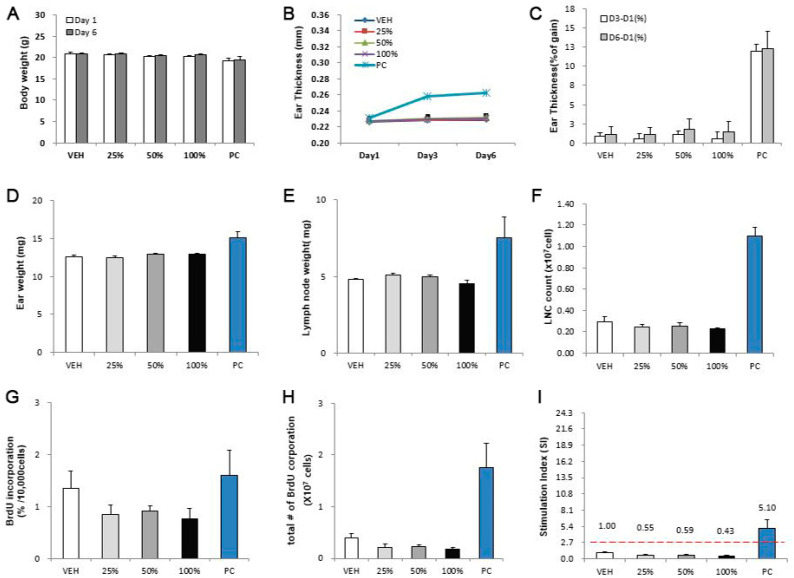
SWCNT skin sensitization test results in LLNA: BrdU-FCM assay. The evaluation parameters were as follows: (**A**) Body weight (g), (**B**) ear thickness (mm), (**C**) ear thickness (% of gain), (**D**) ear weight (mg), (**E**) lymph node weight (mg), (**F**) lymph node cell (LNC) count (×10^7^ cells), (**G**) BrdU incorporation (%/10,000 cells), (**H**) total number of BrdU corporation (×10^7^ cells), (**I**) stimulation index (SI). Data are expressed as mean ± standard deviation values (*n* = 4).

**Figure 5:**
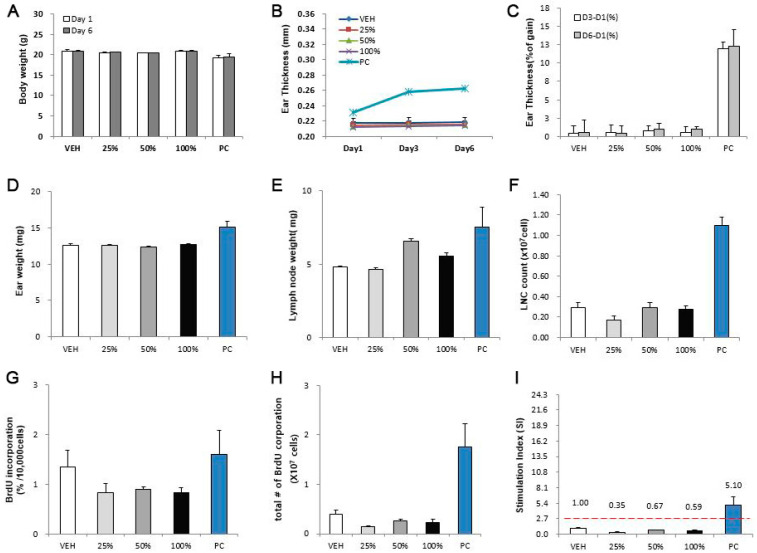
MWCNT skin sensitization test results in LLNA: BrdU-FCM assay. The evaluation parameters were as follows: (**A**) Body weight (g), (**B**) ear thickness (mm), (**C**) ear thickness (% of gain), (**D**) ear weight (mg), (**E**) lymph node weight (mg), (**F**) LNC count (×10^7^ cells), (**G**) BrdU incorporation (%/10,000 cells), (**H**) total number of BrdU corporation (×10^7^ cells), (**I**) stimulation index (SI). Data are expressed as mean ± standard deviation values (*n* = 4).

SWCNTs and MWCNTs were assessed for their skin sensitization potential using the LLNA: BrdU-FCM assay (Figure 4 and Figure 5). Except for the positive control, no significant results were found at any concentration in the CNTs. The SI values of SWCNTs and MWCNTs were less than 2.7, as calculated by flow cytometry.

## 4. Discussion

**Figure 6:**
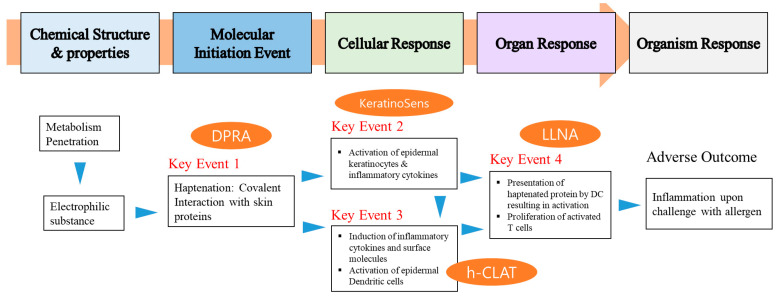
Overview of skin sensitization adverse outcome pathway (AOP).

With the growing emphasis of the 3R principles of reduction, replacement, and refinement of test animals, the use of test animals in toxicity studies in the recent international community has always been a major issue [13]. Animal alternative testing methods have been suggested by various countries and institutions, including the European Union Reference Laboratory for Alternatives to Animal Testing, Interagency Coordinating Committee on the Validation of Alternative Methods, and Japanese Center for the Validation of Alternative Methods. Various studies are being carried out on this subject, and the OECD has approved, enacted, and distributed guidelines for alternative test methods. The OECD TG 442 guidelines can be classified into four key events as follows: Key events 1: Molecular initiation event; key events 2, 3: Cellular response; and key events 4: Organ response based on AOP inducing skin sensitization (Figure 6).

The most basic step to induce skin sensitization depends on the ‘immunogenicity’ of the substance. Nanomaterials are of solid form but have a very small size compared to bulk materials. One dimension has a size of less than 100 nm, and these substances have the potential to induce an immune response through “Haptenation” by binding to carrier proteins in the physiological environment. The CNT used in this study is a substance that is mentioned as an adjuvant candidate and has the potential to induce an immune response by binding to a carrier protein [26]. In addition, it has been reported that when exposed to the body, it penetrates into tissues and induces persistent inflammatory cytokines, as well as a tendency to attract inflammatory cells and lymphocytes to the inflammatory site [23]. Therefore, in order to evaluate the skin sensitization potential of CNTs, we evaluated, by adopting key events 2 and 4, the method of confirming cellular response and organ response in skin sensitization AOP.

The accuracy of the KeratinoSens™ assay for identifying sensitizers was shown to be 77% (155/201), with a sensitivity of 78% (71/91). In addition, laboratory-to-laboratory reproducibility has been reported to be approximately 85% [27,28]. Although there are restrictions on testing for insoluble substances, some research cases have proven that these substances can be evaluated [29,30]. The LLNA: BrdU-FCM test method uses animals, and previously reported studies have suggested the possibility of evaluating nanomaterials. Park et al. conducted an LLNA test using titanium nanomaterials and reported that titanium did not induce sensitization [20].

The two types of CNTs used in our study are insoluble in most solvents and have the characteristic to easily form aggregates. As the major toxicity indicator of nanomaterials, proper dispersion is very important in predicting accurate toxicity, and hence, homogeneous dispersion in solvents is important. We used serum protein to improve the dispersion of CNTs in both tests [31]. In the in vitro test, dispersion was induced using the FBS component contained in the medium. In particular, the mouse serum, as a nanomaterial dispersant used in animal tests, not only induces improvement in large aggregation when inactivated serum obtained from the same species/line was used, but also proved that there were no side effects caused by the serum [32,33].

We report for the first time the sensitization results of CNTs using the KeratinoSens™ and LLNA: BrdU-FCM test methods in this study. In summary, the skin sensitization results for both SWCNTs and MWCNTs, using the two alternative tests, were negative. In our study, IC50 results were established for the KeratinoSens^TM^ test for the first time, which was established based on the mass dose. Cytotoxicity by CNTs is primarily influenced by physical factors related to size such as aspect ratio and length. Asbestos-like forms of CNTs can cause incomplete phagocytosis and induce the formation of granulomas in the mouse pleura through persistent lymphocyte recruitment and cytokine induction in the chronic inflammatory stage [23]. In our study results, it was observed that SWCNTs induced a higher cytotoxicity than MWCNTs at the same concentration. SWCNTs with thinner diameters at smaller lengths appear to induce high cytotoxicity.

In addition, this study is the first alternative test case of SWCNTs evaluated using mice. Ema et al. performed the traditional skin sensitization test, guinea pig maximization test (GPMT), to evaluate CNTs, and reported SWCNTs and MWCNTs as the final nonsensitizing substance [34]. In addition, MWCNTs were evaluated as substances that do not induce sensitization in LLNA tests using mice [35,36]. There are currently a wide variety of types of carbon nanotubes, but in this study, two types of nanotubes were used only. In order to ensure the safety of commercialized CNTs, it will be necessary to accumulate data through more studies.

## 5. Conclusions

In this study, we report for the first time the sensitization results of CNTs using the KeratinoSens™ and LLNA: BrdU-FCM test methods in this study. This study found that both SWCNTs and MWCNTs do not induce skin sensitization for in vitro and in vivo levels. These results suggest that the ARE-Nrf2 Luciferase KeratinoSens™ and LLNA: BrdU-FCM assay may be useful as alternative assays for evaluating the potential of some nanomaterials that can induce skin sensitization. Further studies are needed evaluate the sensitization of nanomaterials more accurately. In addition, it is necessary to establish skin sensitization guidelines for specific nanomaterials based on various studies.

## Figures and Tables

**Table 1:** Characteristics of SWCNTs and MWCNTs.

Characteristic	KeratinoSens™		LLNA: BrdU-FCM	
	SWCNT	MWCNT	SWCNT	MWCNT
Average diameter (nm)	5.97 ± 1.48	12.30 ± 2.18	5.97 ± 1.48	12.30 ± 2.18
Average length (µm)	1	10	1	10
Surface area (m^2^/g)	≥700	216	≥700	216
Zeta potential (mV)				
in DW	−27.40 ± 1.59	−34.99 ± 0.80	−27.40 ± 1.59	−33.99 ± 0.80
in working solution *	−29.23 ± 1.79	−26.99 ± 3.07	−18.80 ± 0.93	−38.38 ± 1.41
CNT purity (%)	≥77	99	≥77	99
Carbon purity (%)	≥90	≥98	≥90	≥98
Endotoxin (EU/mL)	<0.1			

* The working solution was prepared with DW stock (1%) + DMEM, containing 1% FBS in KeratinoSens™ assay. The working solution in LLNA: BrdU-FCM assay was prepared using DW stock (10%) + DMF, containing 3% mouse serum. Data are expressed as mean ± SD, *n* = 6. SWCNTs = single-wall carbon nanotubes, MWCNTs = multi-walled carbon nanotubes, DW = distilled water, EU = endotoxin, DMEM = Dulbecco’s modified Eagle’s medium, FBS = fetal bovine serum, DMF = *N,N*-dimethylformamide.

**Table 2:** SWCNTs and MWCNTs evaluated in KeratinoSens™ assay.

Nanomaterials	CAS RN	Physical Form	KeratinoSens™ Assay Results				
			Imax	EC_1.5_ (µg/mL)	Cell Viability (%) ^a^	IC_50_ (µg/mL)	Classification
SWCNT	308068-56-6	Solid	1.07	>1000	>70	185.90	Negative
MWCNT	308068-56-6	Solid	1.39	>1000	>70	234.98	Negative

^a^ Cell viability (%) at EC_1.5._

## Abbreviations

AOP	Adverse outcome pathway
CNT	Carbon nanotube
DMF	*N,N*-Dimethylformamide
DMEM	Dulbecco’s modified Eagle’s medium
DPRA	Direct peptide reactivity assay
FBS	Fetal bovine serum
FCM	Flow cytometry
IACUC	Institutional Animal Care and Use Committee
LLNA	Local lymph node assay
LNC	Lymph node cells
MFDS	Ministry of Food and Drug Safety
MWCNTs	Multi-walled carbon nanotubes
SI	Stimulation index
OECD	Organization for Economic Cooperation and Development
SWCNTs	Single-wall carbon nanotubes
TEM	Transmission electron microscopy
